# Effect of mean platelet volume and platelet count on the prognosis of branch atheromatous disease

**DOI:** 10.1002/brb3.3509

**Published:** 2024-05-23

**Authors:** Yinglin Liu, Kun Wu, Ronghua Xu, Lanying He, Min Zheng, Jian Wang

**Affiliations:** ^1^ Department of Neurology Chengdu Second People's Hospital Chengdu Sichuan China; ^2^ Department of Laboratory Yibin Sixth People's Hospital Chengdu Sichuan China; ^3^ Department of Laboratory University of Electronic Science and Technology Chengdu Sichuan China

**Keywords:** branch atheromatous disease, early neurological deterioration, mean platelet volume, platelet count, stroke outcome

## Abstract

**Objective:**

The purpose of this study was to investigate the predictive value of mean platelet volume (MPV) and platelet count (PC) in branch atheromatous disease (BAD).

**Methods:**

This retrospective study included 216 patients with BAD‐stroke within 48 h of symptom onset. These patients were divided into good and poor prognosis groups according to their 3‐month modified Rankin scale scores after discharge. Multiple logistic regression analysis was used to evaluate independent predictors of poor prognosis in BAD‐stroke patients. Receiver‐operating characteristic (ROC) analysis was used to estimate the predictive value of MPV and PC on BAD‐stroke.

**Results:**

Our research showed that a higher MPV (aOR, 2.926; 95% CI, 2.040–4.196; *p* < .001) and PC (aOR, 1.013; 95% CI, 1.005–1.020; *p* = .001) were independently associated with poor prognosis after adjustment for confounders. The ROC analysis of MPV for predicting poor prognosis showed that the sensitivity and specificity were 74% and 84.9%, respectively, and that the AUC was .843 (95% CI, .776–.909, *p* < .001). The optimal cut‐off value was 12.35. The incidence of early neurological deterioration (END) was 24.5% (53 of 163), and 66% of patients in the poor prognosis group had END (33 of 50). Multiple logistic regression analyses showed that elevated MPV and PC were associated with the occurrence of END (*p* < .05).

**Conclusion:**

Our results suggested that an elevated MPV and PC may be important in predicting a worse outcome in BAD‐stroke patients. Our study also demonstrated an independent association of MPV and PC with END, which is presumably the main reason for the poor prognosis.

## Graphical abstract

BAD‐stroke patients with high MPV and PC are more likely to develop END and have poor prognosis at 3 months.

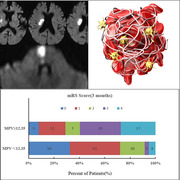

## 1 INTRODUCTION

Branch atheromatous disease (BAD) is a single deep cerebral infarction caused by stenosis or occlusion of the perforator artery orifice (Petrone et al., 2016). BAD‐stroke is a common type of acute ischemic stroke, and its prevalence was reported to be as high as 10.4%–18.3% (Wu et al., 2020). Clinically, BAD primarily manifests as early neurological deterioration (END) dominated by progressive motor deficiency, which often results in a poor prognosis (Oh et al., 2012; Yamamoto et al., 2011). A study found that the incidence of END was as high as 39.4% among BAD‐stroke patients (Sun et al., 2017). In daily practice, END and prediction of the prognosis of BAD‐stroke patients are the most important clinical problems.

Platelets play a crucial role in the development and progression of ischemic stroke because of their involvement in thromboembolism (Greisenegger et al., 2004). Mean platelet volume (MPV) and platelet count (PC) are the two main parameters that reflect platelet function and activities. Platelets with a higher MPV are more functionally and metabolically active, contain more dense granules, and promote further platelet aggregation as well as activation (Muscari et al., 2009; Sadeghi et al., 2020). Previous studies have shown that MPV is significantly increased in patients with coronary artery disease, and patients with high MPV have a 17% higher risk of cardiovascular events compared to those with low MPV (Thakkinstian et al., 2017). Patients with acute ischemic stroke (AIS) have also been shown to have significantly higher MPV. MPV and PC were observed to be independent risk factors for ischemic stroke (Mayda‐Domac et al., 2010; Sadeghi et al., 2020). Previous studies have suggested that MPV is associated with the prognosis of AIS (Du et al., 2016; Greisenegger et al., 2004; Wan & Ma, 2017; Xu et al., 2020; Yang et al., 2019; Ye et al., 2020), although some studies have reported contradictory findings (Du et al., 2016; Mayda‐Domac et al., 2010). The studies of PC have mostly been related to cardiology and showed that PC was an independent risk factor for cardiovascular mortality in acute myocardial infarction (AMI) patients (Goliasch et al., 2013; Ly et al., 2006; Song et al., 2021). However, the few studies on the relationship between PC and the prognosis of BAD‐stroke have reached different conclusions (Mayda‐Domac et al., 2010; Yang et al., 2019; Ye et al., 2020).

At present, there are no notable reports on the relationship between MPV or PC and the development and prognosis of BAD‐stroke. Thus, the principal aim of this study was to explore whether baseline MPV and PC have an impact on the development and prognosis of BAD patients.

## 2 MATERIALS AND METHODS

This retrospective study was approved by the Medical and Health Research Ethics Committee of the Second People's Hospital of Chengdu (Chengdu, China) and adhered to the Declaration of Helsinki. Because it was a retrospective study, informed consent was not required, and all included patient information was anonymous.

### 2.1 Research subjects

We retrospectively collected BAD‐stroke patients at Chengdu Second People's Hospital from January 2020 to February 2022. The inclusion criteria were as follows: (1) patients presenting within 48 h of onset; (2) diffusion‐weighted imaging (Feigin et al., 2021) within 48 h of admission showing BAD‐related stroke; and (3) patients with a modified Rankin scale (mRS) score ≤1 before admission. The exclusion criteria included the following: (1) patients who did not undergo blood cell analysis; (2) patients who did not meet the diagnostic criteria for BAD; (3) patients receiving thrombolytic therapy; (4) patients with various bleeding diseases or coagulation dysfunction; (3) patients with cardiogenic embolism or use of coagulation medication; (4) patients with severe cardiopulmonary, liver, and kidney insufficiency, combined with malignant tumors; (5) patients with incomplete follow‐up at 3‐month poststroke; and (6) patients with incomplete clinical data. All the patients included in the study received 21 consecutive days of double‐antibody therapy and 14‐day intensive lipid‐lowering treatment (atorvastatin 40 mg/day) after admission and were then treated with long‐term single antibody therapy and lipid‐lowering therapy (atorvastatin 20 mg/day) after discharge.

### 2.2 Data collection

Two clinicians reviewed the electronic medical record system from Chengdu Second People's Hospital to collect information, and a data extraction form was designed to record patient information. Basic information included age, sex, hypertension, diabetes, smoking, drinking, history of stroke, and history of taking antiplatelet drugs, and clinical data included time from onset to arrival, blood pressure at admission, random blood sugar, National Institutes of Health Stroke Scale (NIHSS) score at admission, presence of END, infarct site, hospital days, and follow‐up results. These risk factors were evaluated as follows—(1) hypertension: repeated blood pressure measurements of ≥140/90 mmHg or a history of previous hypertension or use of antihypertensive drugs; (2) diabetes: history of previous diabetes or the use of diabetes medications, or more than two measurements of fasting plasma glucose >7.0 mmol/L or random plasma glucose >11.1 mmol/L at the time of admission; (3) smoking: ≥10 cigarettes per day; and (4) drinking: alcohol consumption >2 U/day (Xu et al., 2021). Baseline examinations included routine laboratory tests, such as blood cell, triglyceride (TG), total cholesterol (TC), low‐density lipoprotein (LDL), high‐density lipoprotein (HDL), creatinine, and glycated hemoglobin tests.

### 2.3 MPV and PC determination

Peripheral venous blood samples (used to measure routine blood parameters, coagulation, renal function, etc.) were collected from all included patients in EDTA anticoagulant tubes on admission (before the administration of antiplatelet drugs), stored at room temperature, and submitted for examination within 2 h. All blood samples were analyzed by a Sysmex XN‐9000 automatic blood cell analyzer. Laboratory physicians were responsible for reviewing the results.

### 2.4 BAD and END definitions

BAD was defined as follows: (1) DWI showing that the infarct in the lenticulostriate artery blood supply area involved three levels or more at the horizontal level or lesions extending to the ventral pontine surface in the blood‐supply region of the paramedian pontine artery; and (2) no evidence of responsible vessel stenosis (>50%) or occlusion and embolism (Park et al., 2016; Petrone et al., 2016; Yamamoto et al., 2011). END was defined as an NIHSS score increase of more than two points within 1 week (Park et al., 2016; Wu et al., 2020).

### 2.5 Follow‐up

All the included subjects were followed up 3 months after onset by telephone or face‐to‐face interviews, and the patients were grouped into good prognosis and poor prognosis groups according to the 3‐month mRS score. Poor prognosis at 90 days was defined as an mRS score >2 points.

### 2.6 Statistical analyses

SPSS Version 25.0 software (IBM Corp) was used for statistical analysis. Continuous variables are expressed as the mean ± standard deviation or as the median and interquartile range. Differences among groups were compared using a t test or the rank‐sum test. Categorical data are presented as frequencies (percentages), and the differences among groups were compared using the chi‐squared test or Fisher's exact test. Variables in the univariate analyses (*p* < .10) were included in the multivariate analysis. Receiver‐operating characteristic (ROC) analysis was used to assess the diagnostic value of MPV and PC in predicting prognosis. Statistical significance was set at *p* < .05.

## 3 RESULTS

### 3.1 Baseline characteristics and follow‐up results

**FIGURE 1:**
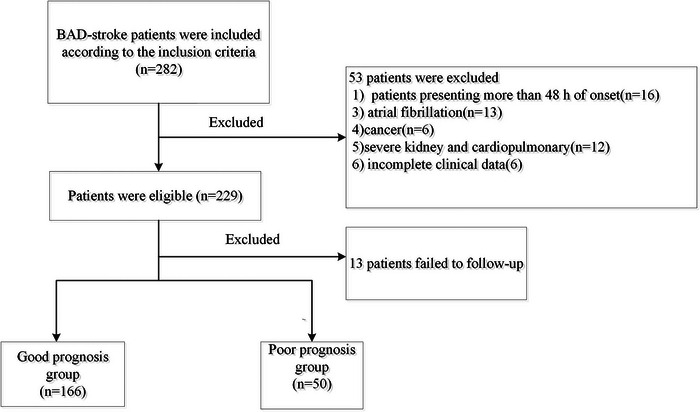
Flowchart of the selection of eligible subjects.

Between January 2020 and February 2022, 282 BAD‐stroke patients within 48 h of stroke onset were enrolled in this study. Fifty‐three patients met the exclusion criteria, and 13 patients were lost to follow‐up. Finally, 216 patients were included in the study. Figure 1 shows the flowchart of the selection of eligible study subjects. At the 3‐month follow‐up, 166 patients (76.8%) had a good prognosis, and 50 patients (23.2%) had a poor prognosis.

### 3.2 Comparison of clinical baseline data in the good and poor prognosis groups

Univariate analysis results showed that MPV and PC were significantly higher in the poor prognosis group than in the good prognosis group (*p* < .05; Table 1). There were also statistically significant differences in sex, NIHSS score at admission, random blood sugar, and creatinine between the good and the poor prognosis groups (*p* < .05; Table 1).

**TABLE 1:** Univariate analysis results of the general and clinical data of branch atheromatous disease (BAD)‐stroke patients in the good and poor prognosis groups.

	Good* (*n* = 166)	Poor* (*n* = 50)	*p*
Age, year	68.0 (57.0,76.0)	66.5 (58,74.5)	.911
Female, sex, *n* (%)	55 (33.1)	25 (50.0)	**.03**
Hypertension, *n* (%)	123 (74.1)	40 (80.0)	.395
Diabetes, *n* (%)	55 (33.1)	16 (32.0)	.881
Smoking, *n* (%)	51 (30.7)	11 (22.0)	.232
Drinking, *n* (%)	26 (15.7)	5 (10.0)	.317
History of ischemic stroke, *n* (%)	3 (1.8)	1 (2.0)	.929
History of taking antiplatelet drugs, *n* (%)	2 (1.2)	1 (2.0)	.548
**Blood pressure at admission**			
SBP, mmHg	152.9 ± 22.0	157.7 ± 22.5	.191
DBP, mmHg	85.0 (76.0,97.0)	89.5 (78.8,97.0)	.584
Arrival time, hours	24.0 (11.0,39.0)	24.0 (12.0,35.3)	.892
NIHSS score at admission, hours	2.5 (2,3)	4.5 (3,6)	<**.001**
Infarct site, *n* (%)			
LSA	103 (62.0)	27 (54.0)	.308
PPA	63 (38.0)	23 (46.0)	
Hospital days, days	12 (10,14)	12 (10.8,14.3)	**.079**
**Laboratory test**			
Random blood sugar, mmol/L	5.6 (4.9,7.4)	6.3 (5.2,10.7)	**.037**
TC, mmol/L	4.78 ± 1.29	4.98 ± 1.66	.378
TG, mmol/L	1.38 (1.03,2.21)	1.43 (.99,1.91)	.472
HDL, mmol/L	1.09 (.90,1.29)	1.13 (.92,1.42)	.520
LDL, mmol/L	2.78 (2.21,3.51)	2.82 (2.25,3.93)	.598
Creatinine, mmol/L	72.0 (62.0,84.0)	63.5 (52.5,72.0)	**.001**
MPV (fL)	10.85 (10.2,11.9)	13.55 (12.0,14.2)	<**.001**
PC (×10^3^/mL)	185.0 (158.0,227.0)	216.5 (187.75,263.75)	**.001**
MPV/PC	0.059 (0.048,0.0733)	0.059 (0.050,0.071)	.780
Glycated hemoglobin (%)	6.1 (5.6,7.4)	6.3 (5.7,8.9)	.241

Abbreviations: Good* means good prognosis group, and poor* means poor prognosis group; DBP, diastolic blood pressure; HDL, high‐density lipoprotein; LDL, low‐density lipoprotein; LSA, lenticulostriate artery; MPV, mean platelet volume; NIHSS, National Institutes of Health Stroke Scale; PC, platelet count; PPA, paramedian pontine artery; SBP, systolic blood pressure; TC, total cholesterol; TG, triglycerides. Bold text emphasizes that the indicator has statistical significance.

### 3.3 Multivariate logistic regression analysis results of factors affecting prognosis

When the factors associated with poor prognosis in univariate analyses (*p* < .10) were entered into multivariate logistic regression analysis, the results showed that the associations of NIHSS score at admission (aOR, 1.870; 95% CI, 1.391–2.514; *p* < .001), MPV (aOR, 2.926; 95% CI, 2.040–4.196; *p* < .001), and PC (aOR, 1.013; 95% CI, 1.005–1.020; *p* = .001) with poor prognosis remained (Table 2).

**TABLE 2:** Multivariate logistic regression analysis of factors influencing poor prognosis.

Risk factors	OR	95% CI	*p*
Sex	.560	.195–1.611	.283
NIHSS score at admission	1.870	1.391–2.514	<**.001**
Random blood sugar, mmol/L	1.108	.973–1.263	.123
Creatinine, mmol/L	.973	.943–1.003	.077
MPV (fL)	2.926	2.040–4.196	<**.001**
PC (×10^3^/mL)	1.013	1.005–1.020	**.001**
Hospital days	1.003	.975–1.031	.850

Abbreviations: END, early neurological deterioration; MPV, mean platelet volume; NIHSS, National Institutes of Health Stroke Scale; PC, platelet count. Bold text emphasizes that the indicator has statistical significance.

### 3.4 Univariate analysis and multivariate logistic regression analysis results of clinical characteristics in the END and no‐END groups

**FIGURE 2:**
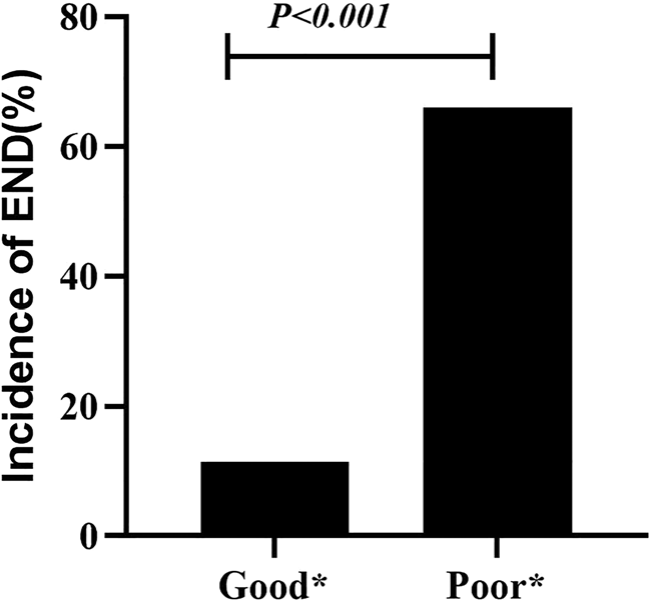
Comparison of the incidence of early neurological deterioration (END) between the good and poor prognosis groups. In the good and poor prognosis groups, the incidence of END was 11.4% (19 of 166) and 66% (33 of 50), respectively. Good* means good prognosis group, and poor* means poor prognosis group.

END was observed in 24.5% (53/216) of all patients and affected 66% (33 of 50) of poor prognosis patients. The incidence of END in the poor prognosis group was significantly higher than that in the good prognosis group (Figure 2). Univariate analysis results showed that there were no significant differences in age, hypertension, diabetes, smoking, drinking, history of ischemic stroke, history of antiplatelet drug use, blood pressure at admission, infarct site, random blood sugar, TG, TC, LDL, HDL, and glycated hemoglobin between the END and non‐END groups (*p* > .05) (Table 3). After adjustment for confounders (sex, hospital days, creatinine, MPV, and PC), multivariate logistic regression analysis showed that the association of creatinine, MPV, and PC with the occurrence of END remained (*p* < .05, Table 4).

**TABLE 3:** Univariate analysis results of the general and clinical data of branch atheromatous disease (BAD)‐stroke patients in the early neurological deterioration (END) and no‐END groups.

	END* (*n* = 53)	No‐END* (*n* = 163)	*p*
Ages, year	67.0 (57.5,74.0)	68.0 (57.0,76.0)	.775
Female, *n* (%)	26 (49.1)	54 (33.1)	**.037**
Hypertension, *n* (%)	42 (79.2)	121 (74.2)	.461
Diabetes, *n* (%)	15 (28.3)	56 (34.4)	.415
Smoking, *n* (%)	15 (28.3)	47 (28.8)	.941
Drinking, *n* (%)	5 (9.4)	26 (16.0)	.240
History of ischemic stroke, *n* (%)	1 (1.9)	3 (1.8)	.983
History of taking antiplatelet drugs, *n* (%)	0 (0)	3 (1.8)	.320
**Blood pressure at admission**			
SBP, mmHg	150.0 (136.5,168.5)	153.0 (141.0,168.0)	.552
DBP, mmHg	84.0 (77.0,96.5)	87.0 (76.0,97.0)	.564
Arriving time, hours	24.0 (12.0,24.0)	24.0 (11.0,48.0)	.585
NIHSS score at admission, hours	3.0 (2.0,4.0)	3.0 (2.0,4.0)	.336
Infarct site, *n* (%)			
LSA	30 (56.6)	100 (61.3)	.540
PPA	23 (43.4)	63 (38.7)	
Hospital days, days	13.0 (11.5,15.0)	12 (10.0,14.0)	**.007**
**Laboratory test**			
Random blood sugar, mmol/L	6.2 (5.0,8.9)	5.6 (4.9,8.2)	.699
TC, mmol/L	4.73 (3.80,5.78)	4.76 (3.83,5.65)	.912
TG, mmol/L	1.43 (1.04,1.91)	1.36 (1.02,2.21)	.814
HDL, mmol/L	1.15 (0.91,1.42)	1.09 (0.90,1.31)	.435
LDL, mmol/L	2.88 (2.23,3.82)	2.78 (2.21,3.52)	.499
Creatinine, mmol/L	63.0 (51.5,74.0)	72.0 (63.0,84.0)	**.002**
MPV (fL)	13.0 (11.2,13.8)	10.90 (10.2,12.0)	**.000**
PC (×10^3^/mL)	208.0 (163.0,257.0)	191.0 (162.0,229.0)	**.084**
MPV/PC	0.060 (0.050,0.078)	0.057 (0.049,0.073)	.463
Glycated hemoglobin (%)	6.2 (5.6,7.6)	6.1 (5.6,7.5)	.870

Abbreviations: DBP, diastolic blood pressure; HDL, high‐density lipoprotein; LDL, low‐density lipoprotein; LSA, lenticulostriate artery; MPV, mean platelet volume; NIHSS, National Institutes of Health Stroke Scale; PC, platelet count; PPA, paramedian pontine artery; SBP, systolic blood pressure; TC, total cholesterol; TG, triglycerides. Bold text emphasizes that the indicator has statistical significance.

**TABLE 4:** Multivariate logistic regression analysis of factors influencing early neurological deterioration (END).

	Multivariate logistic regression analysis		
	OR	95% CI	*p*
Female (%)	.716	.328–1.561	.401
Hospital days	.997	.985–1.008	.550
Creatinine, mmol/L	.975	.954–.998	**.030**
MPV (fL)	1.896	1.494–2.407	<**.001**
PC (×10^3^/mL)	1.006	1.000–1.012	**.043**

Abbreviations: MPV, mean platelet volume; PC, platelet count. Bold text emphasizes that the indicator has statistical significance.

### 3.5 ROC analysis of MPV and PC in predicting poor prognosis

**FIGURE 3:**
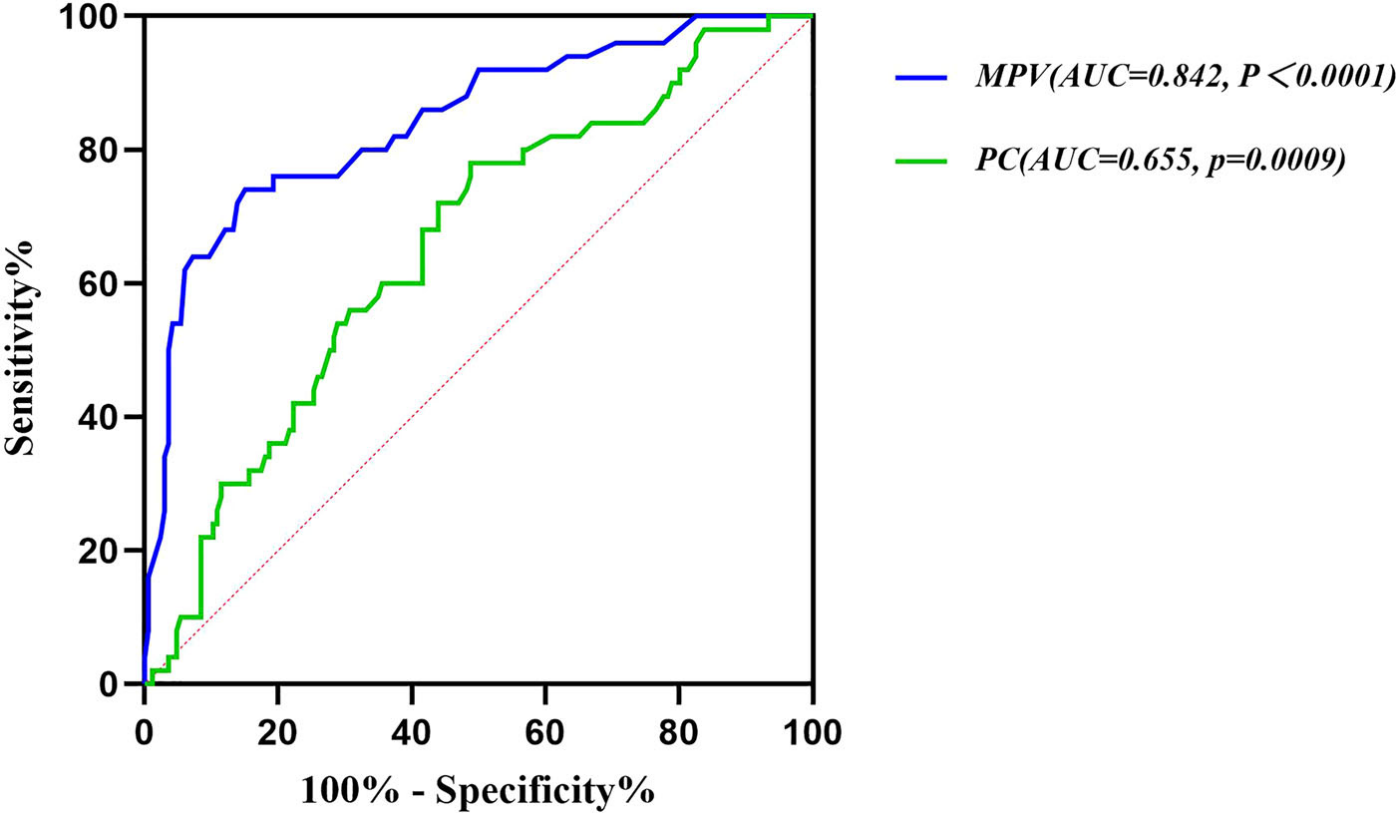
Receiver‐operating characteristic (ROC) analysis of mean platelet volume (MPV) and platelet count (PC) in predicting poor prognosis.

**FIGURE 4:**
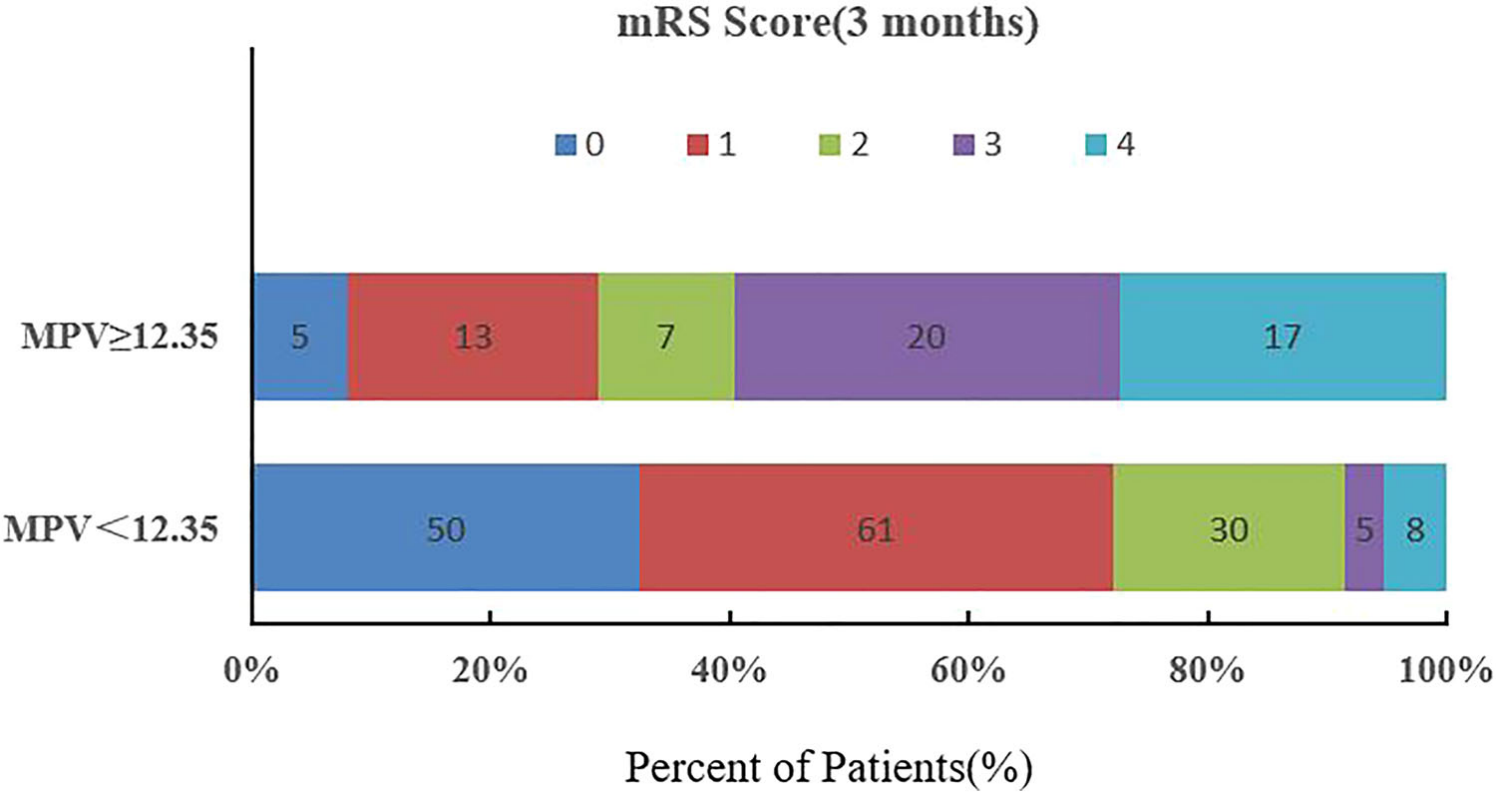
Comparison of the modified Rankin scale (mRS) scores between the good prognosis group and poor prognosis group at 3 months.

The value of MPV and PC in predicting the prognosis of BAD was assessed by ROC curves. The ROC analysis results showed that the sensitivity and specificity of MPV to predict poor prognosis were 74% and 84.9%, respectively, and the AUC was .842 (95% CI, .776–.909, *p* < .0001). The optimal cut‐off value was 12.35. However, the AUC of PC for predicting poor prognosis was .655 (95% CI: .570–.739, *p* = .0009) (Figure 3). A comparison of the mRS scores between the good and poor prognosis groups at 3 months is shown in Figure 4.

## 4 DISCUSSION

In our study, we assessed the association between MPV and PC with the development and prognosis of BAD‐stroke, finding that MPV and PC were closely related to the prognosis of BAD‐stroke. Patients with a higher MPV and PC were more prone to developing END, which occurred in 66% of patients in the poor prognosis group, indicating that there is a strong association between END and poor prognosis in BAD‐stroke.

To our knowledge, there are few analyses of the effect of MPV on stroke subtypes in previous studies, and our study, for the first time, investigated the predictive value of MPV and PC for prognosis in BAD‐stroke patients. MPV was found to be associated with a poor prognosis of BAD‐stroke, and a higher MPV had a negative relationship with 3‐month outcomes of BAD‐stroke patients. The ROC analysis results showed that an MPV ≥12.35 had a sensitivity of 74% in predicting poor prognosis, and the specificity was 84.9%. Previous studies have demonstrated that MPV was associated with poor prognosis in patients with acute cerebral infarction (Greisenegger et al., 2004; Mayda‐Domac et al., 2010; Wan & Ma, 2017; Xu et al., 2020), which was consistent with our findings. There are currently few studies on the relationship between PC and stroke prognosis. In 2019, a subgroup analysis of the China National Stroke Registry (CNSR) II that included 16,842 stroke participants found that PC within the normal range may be a qualified predictor for long‐term recurrent stroke, mortality, and poor functional outcome in ischemic stroke or transient ischemic attack patients (Yang et al., 2019). Ye et al. (2020) showed that patients with cerebral infarction with a PC of 126–225 × 10^9^/L had the lowest mRS scores between 3 months and 1 year after onset. Several studies have previously shown an independent association between PC and cardiovascular mortality or adverse short‐term outcomes in AMI patients (Goliasch et al., 2013; Ly et al., 2006; Nikolsky et al., 2007; Song et al., 2021). However, a study on the prognostic role of MPV and PC in ischemic and hemorrhagic stroke suggested that PC may be a predictor of the prognosis of hemorrhagic stroke but not ischemic stroke (Mayda‐Domac et al., 2010). In addition, Du et al. (2016) revealed that neither MPV nor PC have a significant relationship with the prognosis of hemorrhagic and ischemic strokes. However, Du et al.’s (2016) study defined poor outcomes as mRS scores ≥4 at 30 days after discharge, whereas our study and other studies defined poor outcomes as mRS scores >2 at 90 days after discharge. In addition, MPV and PC were transformed into categorical variables in Du et al.’s (2016) study. However, in our study, MPV and PC were assessed as continuous variables, which could be useful and more reliable in determining the relationship between the prognosis of stroke and platelet parameters. In addition, higher initial NIHSS scores on admission served as risk factors for prognosis in our study, which was similar to Wan and Ma's (2017) study. Higher initial NIHSS scores are suggested to indicate more severe paralysis, so they have a negative correlation with the prognosis of BAD‐stroke.

Clinically, BAD primarily manifests as END, dominated by progressive motor deficiency, and the incidence of END in our study was 24.5%. Patients who experience END often have more severe paralysis. Our study showed that the proportion of patients with END in the poor prognosis group was 66% (33 of 50), which suggested that the presence of END has a close negative correlation with the prognosis of BAD‐stroke (Table 4). Therefore, preventing the occurrence of END can greatly improve the prognosis of patients. The infarct size, infarct location, and NIHSS score on admission have been shown to be significantly associated with END in previous studies (Gokcal et al., 2017; Huang et al., 2019; Jang et al., 2020; Li et al., 2020; Yang et al., 2016). Our study found that the MPV and PC values in the END group were significantly higher than those in the non‐END group, and we demonstrated that elevated MPV and PC are independent risk factors for END in BAD (Table 4). In the PubMed database, only one article concentrated on the relationship between platelets and END in BAD. Oji et al. (2018) retrospectively investigated 64 patients with BAD (17 in the END group and 47 in the non‐END group) and found that the MPV on admission was significantly greater in the END group than in the non‐END group (*p* < .05). Multivariate logistic regression analysis was not performed because of the relatively small number of patients in Oji et al.’s (2018) study, and our study compensates for this shortcoming. A previous study (*n* = 1468) reported that the combination of PC and plasma D‐dimer in cerebral infarction may have more significant prognostic value for END (OR, 3.622; 95% CI, 1.732–7.573) than plasma D‐dimer alone (Liu et al., 2020). Thus, the combination of high MPV and PC values may be an independent biomarker for END in BAD. Clinicians should be alert to the occurrence of END in BAD patients, as this leads to poor prognosis. In addition, creatinine values were found to be associated with END in our study (*p* = .030).

Platelets play an important role in the processes of thrombus formation and atherogenesis (Kamath et al., 2001). The pathological basis of BAD is the blockage of perforating arteries by atherosclerotic plaques (Petrone et al., 2016). Therefore, the number and function of platelets greatly affect the occurrence and development of cerebral infarction (Du et al., 2016). When atherosclerosis occurs, the vessel wall is damaged, which increases the area in contact with platelets, making the vessel wall more prone to thrombosis. An increased MPV leads to more thromboxane A2, the expression of more glycoprotein receptors, such as IIIa and IIb receptors, and stronger chemotaxis, adhesion, and aggregation functions. In addition, a larger MPV can promote the release of more active factors, which exacerbate endothelial cell damage and inflammation, thereby promoting the progression of atherosclerosis and thrombosis (Kamath et al., 2001). We speculate that, due to the abovementioned mechanism, platelets with a larger MPV and a higher PC can cause local thrombus prolongation and repeated embolization of small blood vessels, which lead to END and poor prognosis in BAD patients.

The strengths of our research are as follows: (1) Little is known about the effects of MPV or PC on stroke subtype, and our study explored the relationship between platelet parameters (MPV and PC) and prognosis in BAD‐stroke for the first time. There are also several limitations to our study. First, it was a single‐center retrospective study with a modest sample size, and patients may have had a certain recall bias for functional recovery after 3 months. Second, we did not investigate the compliance of patients taking secondary prevention drugs for cerebral infarction and rehabilitation treatment after discharge, which would have an impact on the patient's 3‐month functional prognosis. Third, PC was collected only at admission, with no record of changes in platelet values during disease. Although platelet indexes are relatively stable (Ilhan et al., 2010; McCabe et al., 2004), research has reported a paradoxical increase in MPV after the initiation of antiplatelet therapy (De Luca et al., 2012).

## 5 CONCLUSION

In conclusion, our findings suggested that elevated MPV and PC may be good predictors of poor prognosis in patients with BAD‐stroke at 3 months. In addition, BAD patients with a high MPV and PC are more likely to develop END, which is the main cause of poor prognosis in BAD patients. For BAD‐stroke patients with MPV values higher than 12.35, more aggressive treatment may be needed to prevent the occurrence of END and improve the outcome of BAD.

## AUTHOR CONTRIBUTIONS

**Yinglin Liu**: Writing—original draft; data curation; formal analysis; conceptualization; methodology; software; visualization. **Kun Wu**: Data curation; writing—original draft; investigation; visualization. **Ronghua Xu**: Project administration; resources; supervision. **Lanying He**: Writing—review and editing; methodology; validation. **Min Zheng**: Formal analysis; project administration; methodology; validation. **Jian Wang**: Writing—review and editing; funding acquisition; methodology; validation; supervision.

## CONFLICT OF INTEREST STATEMENT

The authors have no conflicts of interest to declare.

### PEER REVIEW

The peer review history for this article is available at https://publons.com/publon/10.1002/brb3.3509.

## CONSENT TO PUBLISH

All authors approved the final manuscript and consented to publish.

## Data Availability

All original data can be obtained via email correspondence at 1245374674@qq.com. All charts in this study are presented in the article/Supporting Information section.
